# Do children with left ventricular noncompaction and a noncompaction-to-compaction ratio < 2 have a better prognosis?

**DOI:** 10.1186/s12887-020-02312-5

**Published:** 2020-09-09

**Authors:** Yi Gan, Li Luo, Jie Tian, Lingjuan Liu, Tiewei Lu

**Affiliations:** 1 Department of Cardiology; Ministry of Education Key Laboratory of Child Development and Disorders; National Clinical Research Center for Child Health and Disorders (Chongqing); China International Science and Technology Cooperation base of Child development and Critical Disorders, Children’s Hospital of Chongqing Medical University, Chongqing, People’s Republic of China; 2 Pediatric Department, Maternal and Child Health Hospital of Hubei Province, Tongji Medical College, Huazhong University of Science and Technology, Wuhan, People’s Republic of China; 3 Chongqing Key Laboratory of Pediatrics, Chongqing, People’s Republic of China; 4 Ministry of Education Key Laboratory of Child Development and Disorders, Chongqing, People’s Republic of China

**Keywords:** Cardiomyopathy, Noncompaction-to-compaction ratio, Left ventricular noncompaction

## Abstract

**Background:**

Ultrasonography is commonly used to diagnose left ventricular noncompaction (LVNC). A ratio of noncompacted to compacted myocardium (NC/C ratio) > >2 is often used to diagnose LVNC. However, a large proportion of patients with noncompact myocardium have NC/C < 2, and the prognosis of these patients have not been studied.

**Methods:**

We included children diagnosed with LVNC between 0 and 15 years of age from January 2007 to December 2018. LVNC was diagnosed based on Stöllberger standard when over three trabeculae were found to be associated with the interventricular recesses. A maximal end systolic ratio of noncompacted to compacted layers was NC/C ratio. Outcomes for LVNC subjects with NC/C < 2 and NC/C > 2 were compared using Kaplan-Meier methods.

**Results:**

There were 124 newly diagnosed LVNC cases, classified as isolated (i-LVNC, *n* = 47) or non-isolated (ni-LVNC, *n* = 77) LVNC and NC/C > 2 (*n* = 43) or < 2 (*n* = 81). The median (interquartile range) follow-up duration was 12 (3–30) months for all patients and 16 (6–36) months for survivors. Sixteen patients with i-LVNC died during follow-up. Patients with i-LVNC and NC/C > 2 had worse survival than those with NC/C < 2 (*p* = 0.022).

**Conclusions:**

In conclusion, during a 12-month follow-up, patients with i-LVNC with NC/C < 2 had a benign prognosis and better outcomes than those with NC/C > 2, suggesting that the former could have a more active and routine lifestyle.

Left ventricular noncompaction (LVNC) is increasing in prevalence and is a type of cardiomyopathy classified by an extensive trabeculated myocardium, which was separated into two distinct layers composed of compacted and noncompacted myocardium [1, 2]. The American Heart Association classifies it as a genetic cardiomyopathy caused by arrested myocardial development [2, 3]. LVNC can be detected among individuals of all ages, ranging from fetuses to nonagenarians, and those with left ventricles that are normally sized and well-contracting or dilated and poorly contracting [4, 5]. However, the exact mechanisms underlying the LVNC pathophysiology remain unclear, especially considering the wide age range in which the phenotype presents [6]. Clinical symptoms range from asymptomatic to signs of heart failure, including life-threatening arrhythmias, sudden death, or stroke [7–9]. Despite advancements in medical technology, there are no genetic or imaging modalities that can diagnose LVNC with absolute certainty [10]. Usually, ultrasound is used for diagnosis, and the three most commonly cited echocardiographic criteria include the depths of intertrabecular recesses [11], ratio of noncompacted to compacted myocardium (NC/C ratio) [12], and the number of trabeculations [13] However, none of these diagnostic criteria are optimal in terms of sensitivity and accuracy [7, 14–16].

At our hospital, the diagnosis of LVNC was based on the Stöllberger standard when more than three trabeculae were found to be associated with the interventricular recesses [13]. We also measured the NC/C ratio because we consider it as a critical diagnostic factor, and relatively more patients had an NC/C ratio < 2. Some researchers suggested that overdiagnosis was probably due to the inappropriate implementation of the diagnosis criteria [17]. It is unclear whether patients with an NC/C ratio < 2 have different clinical manifestations and whether they should be treated the same as those with an NC/C ratio > 2. Early diagnosis and correct management of LVNC patients are crucial. Therefore, in this study, we analyzed the late outcomes among children with LVNC (using the Stöllberger criteria) and compared the clinical features as well as patient prognosis based on the NC/C ratio.

## Methods

This observational, single-center, and retrospective study included all children, between 0 and 15 years of age, from the Children’s Hospital of Chongqing Medical University diagnosed with LVNC from January 2007 to December 2018. Medical records were reviewed to document clinical presentations, including symptoms, primary diagnosis, New York Heart Association (NYHA)/Ross classification, associated dysmorphic features, presence of arrhythmia, and a positive family history. Echocardiograms were analyzed for ejection fraction, mitral and tricuspid inflow velocities, ventricular dimensions, and features of congenital heart disease. GE ViVid-7Dimension ultrasonic diagnostic instrument was used and the echoPAC Dimension external digital workstation was equipped with 3 V cardiac probe (GE Healthcare, Boston, MA, USA). All performers who had at least 8 months experience with echocardiography and all records were reevaluated by one experienced cardiologist to confirm the diagnosis. Holter monitors and 12-lead electrocardiograms (ECGs) were examined to detect arrhythmia. In addition, for some patients, cine cardiac magnetic resonance images (MRIs) were analyzed to assist diagnosis. Standardized clinical and echocardiographic data were obtained for patients who were followed regularly. All patients were called by telephone to procure information regarding mortality and reason of death, last functional capacity, syncope, cerebrovascular events, or hospitalization for heart failure. Ethical approval was obtained from the ethics committee of Children’s Hospital of Chongqing Medical University (Approval number: no. 24 of 2015), and informed consents were obtained from parents or guardians on the behalf of participants.

**Fig. 1:**
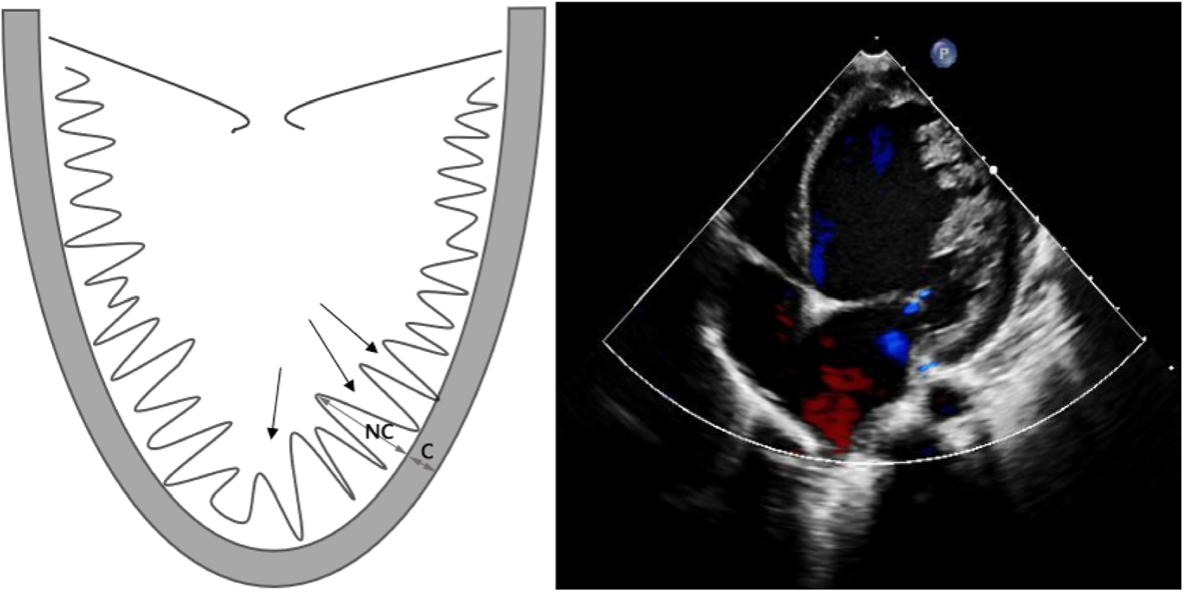
Diagnostic criteria of left ventricular noncompaction, as proposed by Stöllberger: more than three trabeculations in one imaging plane below the level of the papillary muscles. (single-side arrow). End-systolic assessment of noncompaction (NC) layer to compaction(C) layer. (double-headed arrow)

LVNC was diagnosed based on two criteria: (1) more than three trabeculations protruding from the ventricular wall, apically to the papillary muscles and visible in a single image plane as well as (2) intertrabecular spaces perfused from the ventricular cavity and visualized on color Doppler imaging [18]. Further, apical short-axis and four-chamber views was measured in all patients. A maximal end systolic ratio of noncompacted to compacted layers was NC/C ratio [12]. (Fig. 1). Patients who did not have coexisting cardiac abnormalities were diagnosed with isolated LVNC (i-LVNC) [12]. Children with structural heart disease and other myocardium disease were classified as non-isolated LVNC (ni-LVNC). The primary endpoint was patient death. Heart failure classification was defined by its notation in the medical record by the attending cardiologist and/or the NYHA or Ross classifications, if present in the records.

All statistical analyses were performed using the IBM SPSS statistic Version 19(International Business Machines Corporation; Armonk, NY, USA). Images were made using GraphPad Prism Version 6 (GraphPad, San Diego, CA, USA). Continuous variables were expressed as medians (interquartile ranges) and compared using the Wilcoxon sum-rank test, where appropriate. Categorical variables were expressed as proportions and compared using the chi-squared test. Event-free rates were calculated using the Kaplan-Meier methods. Distributions of time to event analyses, categorized by phenotype, were compared using the log rank test. The median follow-up time was defined as the last access to patient data. Owing to the relatively low number of cases, univariable Cox regression analysis was performed to examine the association between variables and outcome of death. Variables evaluated using the univariable regression analysis included age at presentation, presence of heart failure, family history, arrhythmia, ejection fraction, fractional shortening, and NC/C ratio. All *p*-values < 0.05 were considered significant.

## Results

During the 10-year study period, there were 124 newly diagnosed cases of LVNC which included 57 boys and 67 girls. Forty-seven (37.9%) and 77 (62.1%) patients were diagnosed with i-LVNC and ni-LVNC, respectively. Forty (51.9%) patients with ni-LVNC had multiple heart defects, with the most frequent lesions being patent ductus arteriosus, atrial septal defects, ventricular septal defects, or single ventricular or aortic stenosis. Furthermore, 43 (34.7%) patients had an NC/C ratio > 2, and 81 (65.3%) had an NC/C ratio < 2 (Table 1). Additional LVNC was confirmed using characteristic findings of left ventricular angiography in six patients and by using MRI in 10.

**Table 1:** Classification of all LVNC patients (*n* = 124)

	NC/C < 2 (*n* = 81)	NC/C > 2 (*n* = 43)
i-LVNC(*n* = 47)	23 (18.5%)	24 (19.5%)
ni-LVNC(*n* = 77)	58 (46.8%)	19 (15.3%)

*LVNC* left ventricular noncompaction. *NC/C* noncompaction-to-compaction ratio

**Fig. 2:**
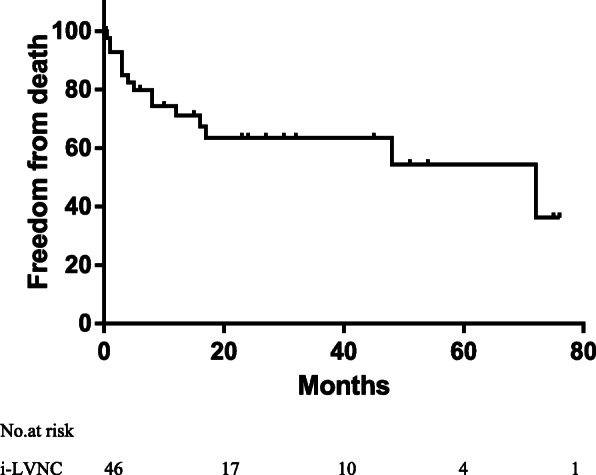
Long-term freedom from death of all 47 i-LVNC subjects. i-LVNC, isolated left ventricular noncompaction

**Fig. 3:**
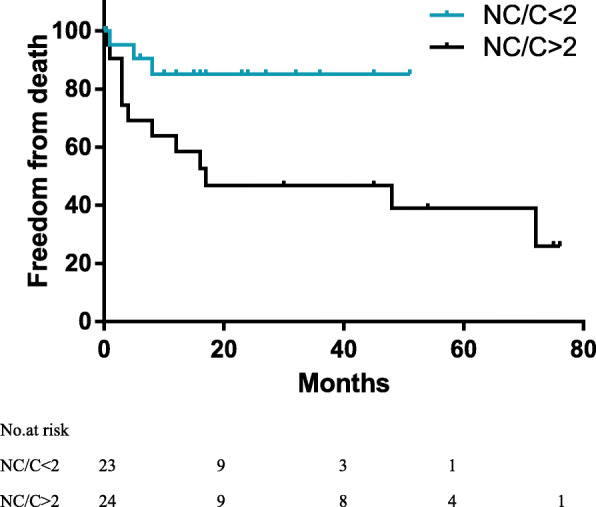
Kaplan-Meier estimate of the cumulative freedom from death to follow-up. The i-LVNC patients with an NC/C ratio > 2 have worse survival than those with an NC/C ratio < 2 (*p* = 0.022). i-LVNC, isolated left ventricular noncompaction; NC/C, noncompaction-to-compaction ratio.

Moreover, 32 patients with ni-LVNC underwent surgery during the follow-up period. Noncompaction was not found after surgery in 18 patients but did not change in the remaining 11 patients. One patient with atrial septal defects showed spontaneous healing but did not show changes in noncompact layer. Additionally, 16 patients with i-LVNC died during follow-up; a patient who drowned unexpectedly was considered lost to follow-up. Eleven (73.3%) of these patients died within 1 year. Freedom from death was 68.1% (95% confidence interval [CI], 52.9–80.9) for patients with i-LVNC. The median (interquartile range) follow-up duration was 12 (3–30) months for all patients and 16 (6–36) for survivors. Figure 2 shows the survival for all cases of i-LVNC. Table 2 shows the association between clinical factors and time to death, as determined by the univariable Cox regression analysis. In this study, no factor was found to significantly affect prognosis. However, the presentation of family history, arrhythmia, and heart failure seemed to show poor prognosis. Patients with NC/C < 2 had NYHA /Ross classification of III/IV, which was significantly lesser than the patients with NC/C > 2 (*p* = 0.029). Significantly more abnormal ECG findings were observed in patients with NC/C > 2 than among those with NC/C < 2 (*p* = 0.01). Left ventricular ejection fraction and fractional shortening were not significantly different between the groups (Table 3). In addition, Kaplan-Meier estimate of cumulative freedom from death to follow-up time revealed that patients with i-LVNC and NC/C ratio > 2 had worse survival than those with NC/C < 2 (Log Rank *p* = 0.022) (Fig. 3).

**Table 2:** Univariable predictors of death after diagnosis of i-LVNC (*n* = 47)

Variable	HR	95% Confidence interval	*p* value
Male	1.001	0.99–1.01	0.845
Presentation with heart failure	1.47	0.33–6.58	0.613
Family history of cardiomyopathy	3.88	0.47–31.85	0.207
Arrhythmia/abnormal findings on electrocardiography	2.03	0.63–6.55	0.236
Baseline EF	1.01	0.97–1.06	0.573
Baseline FS	1.03	0.95–1.12	0.498
NC/C ratio	1.72	0.90–3.29	0.098

*EF* ejection fraction, *FS* fractional shortening, *HR* hazard ratio, *i-LVNC* isolated left ventricular noncompaction, *NC/C ratio* noncompaction-to-compaction ratio

*p* < 0.05 shows statistical significance

**Table 3:** Comparison between NC/C > 2 and NC/C < 2 of patients with i-LVNC

Characteristics	NC/C < 2	NC/C > 2	*p*-value
	*n* = 23	*n* = 24	
Age of diagnosis (months)	7.2 (2.2–34)	6.8 (3.5–44.5)	0.52
0–1(Y)	13 (56.5%)	15 (62.5%)	–
1–15 (Y)	10 (43.5%)	9 (37.5%)	0.68
Sex ratio (female: male)	48:52 (11:12)	50:50 (12:12)	0.88
Weight – median (IQR), kg	6.5 (5.5–12.3)	7.5 (5.4–11.3)	0.57
NYHA class/ Ross at diagnosis – n (%)			
I	5 (21.7%)	4 (16.7%)	–
II	9 (39.1%)	3 (12.55)	–
III	6 (26.1%)	7 (29.2%)	–
IV	3 (13%)	10 (41.7%)	**0.03**
Arrhythmia/abnormal findings on electrocardiography	9 (39.1%)	18 (75%)	**0.01**
Thromboembolic	0 (0%)	1 (4.2%)	0.32
Family history	1 (4.3%)	1 (4.2%)	0.97
LVEF-median (IQR)	46.5 (36.3–51)	35 (31–44.5)	0.07
FS-median (IQR)	22.5 (17.5–26)	16 (14.5–22)	0.07
NC/C ratio: median (IQR)	1.5 (1.4–1.8)	2.4 (2.2–3.1)	**< 0.01**
Number of deaths	3	12^a^	**0.01**
Median (IQR) duration of follow-up, months	12 (6–24)	10 (1–42.3)	0.69

*i-LVNC* isolated left ventricular noncompaction, *IQR* interquartile range, *LVEF* left ventricular ejection fraction, *FS* fractional shortening, *NC/C ratio* noncompaction-to-compaction ratio, *NYHA* New York Heart Association

Data are expressed as median (IQR) or as number (percentage). *p* < 0.05 shows statistical significance

^a^Another patient death occurred, which was not included in the analysis

## Discussion

We present the first retrospective study on NC/C < 2 in LVNC patients so far, and found that patients with i-LVNC and NC/C < 2 had good prognosis than those with NC/C < 2 (*p* = 0.022) and three cases of deaths suggested that i-LVNC patients with NC/C < 2 were not always benign. Over the past decade, there have been remarkable technological advances in image resolution in 2D echocardiography, leading to better diagnosis of LVNC. This study reviewed all hospitalized patients diagnosed with LVNC in the past 10 years and examined late outcomes in these patients based on the NC/C ratio.

Left ventricular noncompaction is characterized by trabeculations in the inner layer of the myocardium and a thinner than usual external compact myocardial layer. This abnormality is often associated with other congenital cardiac defects, such as ventricular defects and aortic constriction [19, 20]. Furthermore, LVNC may be more common in children with congenital heart disease [21]. In our study, more than half of the patients with LVNC had congenital heart disease; incomplete trabeculation was the secondary change following other myocardial diseases [22]. There are three commonly cited echocardiographic definitions to identify LVNC, including the depths of intertrabecular recesses, NC/C ratio, and number of trabeculations. All of them have been widely used in clinical settings, with them having known limitations [23, 24]. Kohli et al. studied these criteria and found that there was a poor correlation between them, with only 29.8% of patients fulfilling all three criteria; these parameters were also found to be highly subjective [24]. In addition, the diagnosis of LVNC is prone to interobserver variability. Even when using the same definition, experienced echocardiographers from different laboratories disagreed in 35% of cases, and after mutual review, 11% of cases remained questionable [25]. In our study, diagnosis was based on echocardiographic criteria proposed by Stöllberger [13]. Considering that the NC/C > 2 standard proposed by Jenni [12] has also been widely used, we combined two different diagnostic criteria to analyze the patients in our study. In most of the research, patients with NC/C < 2 were generally excluded from LVNC and has not been systematically studied so far [12]. However, we found that patients with LVNC< 2 accounted for about half of the patient group in this research. Some researchers have suggested that technological advances in image resolution have led to a more intricate visualization of the left ventricular myocardium, resulting in overdiagnosis of LVNC [26]. The important question posed by this study was whether the high proportion of patients fulfilling the current diagnostic criteria for LVNC with an NC/C ratio < 2 was explained by a genuine congenital abnormality or an exaggeration of the normal trabeculation patterns.

According to our classification, more than half of patients were ni-LVNC with cardiac structural changes. Noncompaction was no longer present after reversal of structural heart defects in some patients. Genetic mutation(s) and nongenetic factors, such as loading conditions (volume/pressure load), contribute to ventricular remodeling (concentric remodeling, concentric/eccentric hypertrophy) and the myocardial phenotype seen in LVNC [23, 24]. Noncompaction in these cases may be attributed to the high-pressure exposure of the ventricle, causing the changes to disappear after treatment of the primary disease. This is consistent with the findings of a study by Robert H. Andersen, which indicated that increased cardiac afterload could cause transient noncompaction [22]. However, noncompaction with structural heart diseases owing to genetic abnormalities may persist after treatment. It remains to be fully determined whether LVNC is a physiological or a pathological phenotype of the myocardium. As LVNC may have an important impact on morbidity and mortality, differentiating between variants and LVNC is important, and a reliable diagnosis is crucial.

The prognosis of patients with ni-LVNC is more affected by its concomitant disease, so that we analyzed the clinical characteristics and prognosis of patients with i-LVNC independently. We found that freedom from death was 68.1% for patients with i-LVNC in a median follow-up period of 12 (3–30) months. This result is consistent with the reported 10-year transplant-free survival rate (60–86%) in studies from Toronto and Texas [27, 28]; however, our rate was lower than the 93–95% obtained in studies from Japan and Cincinnati [6, 29]. These differences are likely due to backgrounds of the study patients. Lower mortality in our study may have occurred because we classified patients with LVNC and DCM into ni-LVNC, which have the prognosis similar to that of pure DCM [6]. Furthermore, outcomes are generally better among children with i-LVNC than among those with LVNC with associated cardiomyopathies [6]. The lower mortality in the Japanese study might be reflective of a higher proportion of asymptomatic cases, which is due to the establishment of systematic childhood screening programs in Japan. Additionally, the lower mortality in Cincinnati may be due to the exclusion of patients with arrhythmias. Based on a recent retrospective single institution report, arrhythmias are a significant factor in predicting poor patient outcomes [30]. In our study, most children with i-LVNC died within 1 year of diagnosis, which is consistent with results of a previous study [31]. Cox regression analysis revealed no significant prognostic factors, consistent with findings of another single-center study [32]. However, several studies have demonstrated that younger age at diagnosis, higher immediate left ventricular end diastolic diameter, and noncompact segments, as seen in ECG were associated with poor prognosis [12]. As in other studies, the severity of the systolic dysfunction was the most important predictor of survival at any time during follow-up [31]. Consistent with that finding, the NYHA class of most patients who died (73.3%) was III/IV. The lack of statistical significance for this result may be related to the small number of patients who had follow-up.

The number of patients in the two groups was similar, making it impossible to ignore patients with an NC/C ratio < 2. Although baseline echocardiographic measurements were similar among the groups, abnormal findings on ECG and occurrence of thrombo-embolic events was higher among patients with NC/C > 2 than among patients with NC/C < 2. Multiple modes of LVNC inheritance have been described in the literature; X-linked recessive or autosomal dominant are the most common, while autosomal recessive and mitochondrial inheritance have also been described [27]. In our study, two (4.3%) patients with i-LVNC had a positive family history of cardiomyopathy, which was much lower than 16–44% reported in published studies [27, 29, 31]. This discrepancy may be related to the lack of understanding of the disease, inadequate screening for immediate family members, and unpopular nature of the domestic gene test. Routine genetic testing is critical for establishing a genotype–phenotype correlation and moving to the next step, which involves the genetically confirmed diagnosis of LVNC [23]. Long-term outcomes were much better for children with i-LVNC with an NC/C ratio < 2 compared to those with NC/C > 2, according to the Kaplan-Meier estimate. The mortality rate was much higher among patients with NC/C > 2 than among patients with NC/C < 2; there are two probable explanations for this result. First, the NC/C ratio might be related to disease severity, indicating that the low mortality in this group may reflect a high proportion of asymptomatic cases because of technological advances in image resolution. Some studies revealed that the NC/C ratio was an independent predictor of left ventricular systolic dysfunction [33]. However, in a study that included a follow-up period of 7.2 years, heart transplantation and death were not associated with the NC/C ratio. Moreover, the NC/C ratio has a specific mutation rate, and the reproducibility of measurements is poor [16]. Second, technological advances in image resolution have led to a more intricate visualization of the left ventricular myocardium, resulting in overdiagnosis of LVNC [17]. However, 3 of 23 patients with an NC/C ratio < 2 died, suggesting that patients with this NC/C ratio do not always have benign outcomes or better prognosis. Most studies excluded the NC/C patients from the study, and the treatment of these patients was often neglected [12, 32, 33]. According to our study, we suggest that this group of patients also need to be followed up regularly, and the interval can be relatively extended, so that the prognosis can be accurately determined by long-term follow-up evaluation. Using MRI, genetic testing, and clinical data examinations, further clarification is required regarding overdiagnosis of LVNC in patients with an NC/C ratio < 2.

LVNC is a rare disease in children and our data, which was a pilot study from a single center have local characteristics that can represent disease characteristics in southwest China. However, larger multicenter studies are needed to validate these preliminary findings and generate more conclusive diagnostic and prognostic data. Outcomes from the present study cannot be generalized to children who have noncompaction with other phenotypes, those without symptoms diagnosed during routine family screening, or those diagnosed using genetic testing. The exact reason of death for some patients were not available because they were followed up by phone call. Not all patients underwent genetic and mitochondrial testing during the study period, and the lack of availability of ultrasound several years ago led to an underestimation of the actual morbidity and missed family histories. The limited number of cases did not allow for multivariable analysis to be conducted. Because echocardiographic diagnosis of LVNC was partly subjective, the lack of a central core echocardiogram laboratory in this study was a limitation, which might have resulted in the under-or overdiagnosis of LVNC in our cohort. Only a small number of patients underwent cardiac MRI, which is potentially a more effective modality for diagnosing LVNC.

## Conclusions

Our study indicated that ultrasonic examination was very useful; however, MRI and genetic testing were also critical for confirming diagnosis. During a 12-month follow-up, we found that patients with i-LVNC with an NC/C ratio < 2 had better prognosis and better outcome than those with an NC/C ratio > 2, suggesting that the former can have a more active and routine lifestyle. However, 3 out of 23 patients with the NC/C < 2 phenotype died after the diagnosis, suggesting that a variation exists among these patients and that caution should be exercised when treating these patients.

## Data Availability

The datasets used and/or analyzed during the current study are available from the corresponding author on reasonable request.

## Abbreviations

LVNC: Left ventricular noncompaction
NC/C: Noncompaction to compaction ratio
DCM: Dilated cardiomyopathy
NYHA: New York Heart Association
